# Effectiveness of Combined Copying Skills Training and Pharmacological Therapy for Patients with Migraine

**DOI:** 10.5539/gjhs.v8n6p179

**Published:** 2015-11-05

**Authors:** Zahra Rashid-Tavalai, Nour-Mohammad Bakhshani, Hamed Amirifard, Maryam Lashkaripour

**Affiliations:** 1 Student Scientific Research Center, Zahedan University of Medical sciences, Department of Clinical Psychology, Zahedan, Iran; 2 Children and Adolescents’ Health Research Center, Zahedan University of Medical Sciences, Department of Clinical Psychology, Zahedan, Iran; 3 Department of medicine, Zahedan University of Medical sciences, Zahedan, Iran; 4 Department of Psychiatry, University of Medical sciences, Zahedan, Iran

**Keywords:** coping skills training, combined therapy, pharmacotherapy, quality of life, self-efficacy, migraine

## Abstract

Headache is one of the most common complaints in neurological clinics. The current study carried out to determine the benefits of combined Coping Skills Training (CST) and Pharmacotherapy (Ph) for patients with migraine. Forty patients with migraine recruited from the outpatient clinics of Zahedan University of Medical Sciences(Iran) and randomly assigned to one of two treatment groups: the first group received combined coping skills training (CST) and pharmacotherapy(Ph); and the second group received the pharmacotherapy alone(Ph). Five patients due to lack of regular presence or filling out the questionnaires excluded from the study. Finally, the results of 35 subjects were analyzed. Data collection was done using the World Health Organization Quality of Life Questionnaire, General Self-Efficacy Scale-Sherer, Ways of Coping Questionnaire and Migraine Headache Index. The results of ANCOVA on post-test, after controlling the pre-test scores, suggested a significant difference in self-efficacy scores between CST+Ph and Ph groups. Moreover, results of ANCOVA did not show significant differences between the two groups in the scores of pain severity, quality of life, and the use of coping strategies. Findings of the present study indicated that coping-skills training, as a psychological intervention, improved self-efficacy. Further longitudinal studies are needed to confirm this conclusion.

## 1. Introduction

Headache is a common complaint among individuals who referred to neurological clinics, and Migraine is the most common type of headaches (Kasper & Harrison, 2005). Severe and recurring headaches limit daily life activities, impair the quality of life and reduce productivity (Kurt & Kaplan, 2008). Due to age, sexuality, ethnicity and race of individuals, the prevalence rate of migraine reported differently. In a study, the prevalence rates of migraine have been reported about 6% and 18% in men and women, respectively (Burton, Landy, Down, & Runken, 2009). Kurt and Kaplan (2008) reported that 18% of general population suffered from migraine. Although the primary cause(s) of migraine is unknown, three different mechanisms namely cardiovascular, neurological and neurological inflammation have been attributed to the development of migraine (Silva et al., 2006).

Migraine considered as one of the most debilitating chronic problem in the world (Wright, 2008). In addition to physical complications, it declines the quality of life and performance, disturbs daily life activities (Buse, Rupnow, & Lipton, 2009). The quality of life is defined as the personal conscious of his/her life satisfaction. It is believed that suffering from a chronic disease significantly affects the patient’s general perception of life satisfaction (Rejeski & Mihalko, 2001). Low quality of life, and refrain from personal activities affect the person’s emotional life and decrease individual’s self-efficacy. According to Bandura’s theory, Self-efficacy refers to one’s belief in one’s ability to do successfully a specific behavior in specific situations (Bandura, Barbaranelli, Caprara, & Pastorelli, 1996).

Considering the role of psychological factors, especially stress, in developing and intensifying migraine headaches (Davis, Holm, Myers, & Suda, 1998), psychotherapy has been seriously taken into consideration as an important standard treatment since 1970 (Gauthier, Iyers, & Carrier, 1996). Basically, pain is a complicated psychological phenomenon that people experience and evaluate it differently (Jensen, 1999; Turner, 2000). It is believed that coping style acts as a moderating variable between stressful event and disorders (Carver et al., 1993). Coping style (strategy) refers to intellectual, emotional, and behavioral activities used to deal with a stressor. Two common coping methods are: (a) problem-oriented and (b) emotion-oriented techniques. Problem-oriented coping skills concentrate on the problem or the situation that caused the stress. While, emotion-oriented copings refers to the strategies used to handle feelings of distress (Lazarus and Folkman., 1984). Effective and more appropriate coping with pain and problematic situations empower individuals’ self-efficacy (Hursey, Rains, & Penzin, 2005).

In several studies, the effectiveness of psychological interventions (coping skills training) has been reported, including knee pain (Keefe et al., 1996), asthma (Velsor-Friedrich et al., 2012), chest pain (Keefe et al., 2011), type 1 diabetes (Grey et al., 2009), cardiovascular disease (Williams, Brenner, & Helrns, 2009), and prostate cancer (Campbell et al., 2007). Some studies, also, indicated that coping-skills training increased self-efficacy in the patients with type 2 diabetes (Chesla et al., 2013), cancer (Porter, 2011; Lambert, 2012; Lambert, 2013), and brain injuries (Backhaus, Ibarra, Klyce, Trexler, & Malec, 2010). Although coping skills training, as an effective method for prevention and treatment of psychological and Psychophysiological disorders, used widely in the world but we have not found sufficient data on the effectiveness of combined coping skills training and pharmacotherapy (CST+Ph) for Iranian patients with migraine. Therefore, the current study was conducted to investigate whether combined therapy (CST+Ph) has advantages over pharmacotherapy alone for patients with migraine.

## 2. Materials and Methods

### 2.1 Participants and Procedure

This study as a prospective, randomized and controlled clinical trial was done during 2014 in Zahedan, Iran. The sample was made of forty patients aged 18-50 years with migraine diagnosed by a neurologist and a psychiatrist using HIS diagnostic criteria. All of participants recruited from patients who referred to neurological clinics of Zahedan University of Medical Sciences. The participants were recruited regarding to following inclusion and exclusion criteria:

Inclusion criteria: a) diagnosis of headache (migraine) meeting the International Headache Society criteria (The International Classification of Headache Disorders, 3rd edition); b) chronic headache (experiencing migraine attack about 15 or more days per month for more than 3 months); c) receiving regular pharmacotherapy (analgesics and prophylactic) for headache; d) aged 18-50 years) competency to participate and give informed consent, and f) at least primary school educational level.

Exclusion criteria: participants were excluded for any of the following reasons: a) psychotic disorders, mood disorders, personality disorders and organic brain damages; b) use of any additional treatment for migraine during the study period; c) not willing to continue the treatment program or answering to the questionnaires incompletely.

The participants were randomly assigned into two treatment groups: the first group received coping skills training and pharmacotherapy (CST+Ph), and the second group only received the pharmacotherapy (Ph). Subjects in CST+Ph group participated in seven sessions that held nearly 2 hours per week. CST program presented by a psychologist to the patients in seven -weekly-sessions including: (1) introducing the program and group members, explaining the rules of programme, and administrating the pretests; (2) discussing the symptoms of migraine and variables related to it; (3) explaining the relationship between thoughts and feelings, ways to recognize irrational thoughts and processing errors, training how to re-evaluate and challenge the dysfunctional thoughts; (4 and 5) discussing the role of stress in the onset and maintenance of migraine, and relaxation training; (6) problem solving skill training as well as relaxation training; (7) communication skills and relaxation training.

### 2.2 Instruments

For assessment of patients before and after treatment programs, 4 tools were used including (1) World Health Organization Quality of Life Questionnaire, (2) General Self-Efficacy Scale-Sherer, (3) Ways of Coping Questionnaire and (4) Migraine Headache Index.

The World Health Organization Quality of Life Questionnaire: WHOQOL includes 26 items derived from its 100-item version. This questionnaire measures four wide areas including physical health, psychological health, social relations, and environment. In addition, it also can assess general health. Items of the questionnaire are evaluated on a 5-point scale (very bad, bad, average, good, and very good). Higher score indicates better quality of life. According to Skevington et al., discriminant validity, content validity and internal validity of WHOQOL are 0.8, 0.76, 0.66, and 0.8, respectively (Skevington et al., 2004).

The General Self-Efficacy Scale: The original 23-item scale was developed by Sherer et al. The original scale assesses 2 factors: general self-efficacy and social-self efficacy (Sherer et al., 1982). In the present study the 17-item General Self-Efficacy Scale (Magaletta & Oliver., 1999) was used. The short form of General Self- Efficacy is a reliable and valid scale (Mohammad Amini, Aghayusefi, & Ebrahimi, 2008).

The Ways of Coping Questionnaire (WOCQ): WOCQ contains 66 items that evaluate 8 factors including: Confrontive Coping, Distancing, Self-Control, Seeking Social Support, Accepting Responsibility, Escape/Avoidance, Planful Problem-Solving, and Positive Appraisal. The subjects respond to the items of WOCQ on a 4-point scale that ranges from “I don’t do this at all” (0) to “I do this a lot” (3). The reliability and validity of this questionnaire have been confirmed by Vahedi (2000), Padyab and Ghazinour (2009).

The Migraine Headache Index (MHI): MHI designed and introduced by Hamedanizadeh et al. (Hamedanizadeh, Mahmoudzadeh, Ebadi, & AsadZandi, 2010). They used content validity method to determine the validity of this inventory. For this purpose, the MHI was given to 20 professors to evaluate its content validity and according to their comments, some modifications were done. In order to measure the reliability of this instrument, test-retest method was used and the results confirmed its reliability(r=0.95) (Hamedanizadeh, Mahmoudzadeh, Ebadi, & Asad Zandi, 2010).

## 3. Results

Five patients were excluded from study due to lack of regular attendance at treatment sessions or not responding fully to the questionnaires; therefore, the data of 35 patients were analyzed. Table 1 shows the demographic characteristics of participants by treatment group. The majority of participants in both treatment groups were female.

**Table 1:** Demographic characteristics of participants

Variable	CST+Ph group	Ph group

	F (%)	F (%)
**Gender**		
*Female*	15(83.3)	13(76.5)
*Male*	3(16.7)	4(23.5)
**Education**		
*<high school*	5(27.8)	6(35.3)
*High school*	1(5.6)	3(17.6)
*B.S*	10(55.6)	6(35.3)
*M.A*	2(11.1)	1(5.9)
*Ph.D.*	0(0)	1(5.9)

Table 2 provides the pre and post- tests mean scores of pain severity, quality of life, self-efficacy, emotion-oriented and problem-oriented strategies by treatment group.

**Table 2:** Mean and standard deviation of variables by group

Variable	CST+Ph group	Ph group

M (SD)	M (SD)
***Pain Intensity***		
*Pre-test*	8.17(2.31)	7.47(2.03)
*Post-test*	6.78(2.34)	6.71(2.77)
***Self-efficacy***		
*Pre-test*	81.61(13.38)	78.82(15.28)
*Post-test*	81.05(15.86)	76.65(14.88)
***Quality of life***		
*Pre-test*	56.83(9.86)	57.12(10.69)
*Post-test*	61.72(10.85)	56.94(9.61)
***Emotional-oriented strategies***		
*Pre-test*	32.72(10.54)	33.82(9.53)
*Post-test*	34.72(11.04)	34.47(13.15)
***Problem-oriented Strategies***		
*Pre-test*	35.33(13.27)	31.59(7.04)
*Post-test*	35.17(13.07)	31.35(13.41)

M: Mean and SD: Standard Deviation.

The results of Levine’s test showed that the variances of dependent variables were equal (assumption of homogeneity of variances) and also dependent variables were normally distributed in the population. Table 3 shows summary of results of ANCOVA for effectiveness of CST+Ph for patients with migraine. The predicted main effects of pre-tests for all variables were significant (Table 3). The results of ANCOVA [between-subjects factor: treatment group (CST+Ph, Ph); covariate: pre-test] on post- test, after controlling the effect of pre-test scores, revealed that main effect of treatment group on self-efficacy was significant, F= 5.65, p = .02, ηp2 = .15. The main effects of treatment groups on pain severity, F= 1.3, p = .26, ηp2 = .04, quality of life, F= .48, p = .49, ηp2 = .02, using Emotional-oriented strategies F= .098, p = .76, ηp2 = .003 and Problem-oriented Strategies, F= .04, p = .85, ηp2 = .001, were not significant.

**Table 3:** The results of ANCOVA for the effectiveness of CST+Ph on dependent variables

Dependent Variable	Source	Sum of Squares	df	Mean Square	F	*p*	Partial Eta Squared
*Pain Intensity*	Pretest Group	141.68 2.96	1 1	141.68 2.96	62.15 1.3	0.000 0.26	0.66 0.04
*Self-efficacy*	Pretest Group	2241.9 219.34	1 1	2241.9 219.34	58.01 5.65	0.000 0.023	0.64 0.15
*Quality of life*	Pretest Group	5969.73 27.83	1 1	5969.73 27.83	103.42 0.48	0.000 0.049	0.76 0.02
*Emotional-oriented strategies*	Pretest Group	1742.19 9.54	1 1	1742.19 9.54	17.97 0.098	0.000 0.076	0.36 0.003
*Problem-oriented Strategies*	Pretest Group	2692.17 3.65	1 1	2692.17 3.65	27.88 0.04	0.000 0.85	0.47 0.001

## 4. Discussion

This study aimed to examine whether combined therapy (CST+Ph) has advantages over pharmacotherapy alone for patients with migraine. Results showed that, after controlling for the effect of pre-tests on post-tests, combined therapy increased the self – efficacy, but there were no significant differences between two groups on post intervention scores on the pain severity, quality of life, using Emotional-oriented and Problem-oriented Strategies. The finding of current study related to significant effect of CST on self-efficacy is consistent with findings of Chesla et al. (2013), Porter et al. (2011), Lambert et al. (2012, 2013). It can be said that coping skills training progrme including self-awareness, stress management, dysfunctional thoughts identification, problem solving, anger control, effective relationship, and assertiveness help the patients to recognize, challenge and change their dysfunctional thoughts.

In addition, results of current study showed no significant difference between CST+Ph and Ph alone in using problem and emotion-oriented strategies. Thus, inconsistent with the findings of some previous studies (Shoaakazemi, 2013; Franken, 2001; Litt, 2003) our study did not find significant advantages for CST+Ph over Ph alone. No significant difference between the two groups in problem-oriented strategies may be attributed to the fact that any changing coping style requires long time for practicing acquired skills. In addition, patients may experience their coping style as an adaptive method to solve the problems (Sinha, 2001) and this is a reason for them to stick their old coping style.

Although, severity of pain scores decreased after CST+Ph, but this is not a significant decrease compared with Ph alone. Similar to our results, several studies (Rinaldi et al., 2006; Beckhaus, Ibarra, Klyce, Trexler, & Malec, 2010) showed that psychological treatments such as relaxation, cognitive-behavioral therapy, biofeedback, coping-skills training and educational interventions did not produce significant reduction in frequency of signs and symptoms of the physical diseases. This finding is inconsistent with the findings of reported by Keefe et al., (1996); Keefe et al., (2011) and Porter et al., (2011).

Additionally, inconsistent with the findings of other researchers such as Campbell (2007), Rinaldi (2006) and Grey (2000). The current study did not demonstrate significant difference between SCT+Ph and Ph alone groups on scores of patients’ quality of life. However, it can be said that the quality of life is a complicated and multi-facet concept that depends not only on patients’ mental perception but also related to their physical and social conditions. Thus, improving quality of life requires long-term and comprehensive intervention, in which not only the psychological aspect but also physical, social, and environmental dimensions of individuals must be considered in treatment programs.

Generally, while our study has provide useful information about treatment of migraine using CST and Ph, it has several limitations that must be acknowledged, including the small sample size, short study duration without follow up and lack of fully similar drug doses for all patients.
